# The effects of safinamide according to gender in Chinese parkinsonian patients

**DOI:** 10.1038/s41598-023-48067-8

**Published:** 2023-11-23

**Authors:** M. T. Pellecchia, M. Picillo, M. C. Russillo, V. Andreozzi, C. Oliveros, C. Cattaneo

**Affiliations:** 1 Neuroscience Section, Department of Medicine, Surgery and Dentistry “Scuola Medica Salernitana”, University of Salerno, 84131 Salerno, Italy; 2 Medical Department, Zambon SpA, Bresso, Italy

**Keywords:** Neuroscience, Neurology

## Abstract

The incidence and prevalence of Parkinson’s disease (PD) is expected to raise dramatically over the next decades. Gender-related differences are not yet widely recognized, particularly regarding the response to dopaminergic medications. To analyse gender differences in the clinical effects of safinamide, compared to placebo, in Chinese PD patients of the pivotal XINDI trial. The XINDI study was a phase III, randomized, double-blind, placebo-controlled, multicenter trial. Patients were followed for 16 weeks receiving safinamide or placebo as add-on to levodopa. The primary efficacy endpoint was the change in the mean total daily OFF time. Secondary efficacy endpoints included total daily ON time, ON time with no/non-troublesome dyskinesia, Unified Parkinson’s Disease Rating Scale and Parkinson's Disease Questionnaire-39 items. A post-hoc analysis was performed to describe the efficacy of safinamide in both genders on motor symptoms, motor fluctuations and quality of life. 128 (42%) out of 305 patients enrolled were women and 177 (58%) men. Our additional analyses of the XINDI study have shown that safinamide, compared to placebo, was associated with improvements in motor symptoms, motor fluctuations and quality of life in both genders, with some differences in the response that did not reach statistical significance, possibly due to sample size limitation and post-hoc design of the study. The changes from baseline at week 16 were > 50% higher in the females compared to males for the total daily OFF time (− 1.149 h vs − 0.764 h in males), the total daily ON time (1.283 h vs 0.441 h in males), the UPDRS total score (− 8.300 points vs − 5.253 points in males) and the UPDRS part II score (− 2.574 points vs − 1.016 points in males). The changes from baseline at week 16 were higher in the females compared to males in the “ADL” domain (− 6.965 points vs − 5.772 points in males), the “Emotional well-being” domain (− 6.243 points vs − 4.203 in males), the “Stigma” domain (− 6.185 points vs − 4.913 points in males) and the “Bodily discomfort” domain (− 5.196 points vs 1.099 points in males), while were higher in males in the “Mobility” score (− 6.523 points vs − 4.961 points in females) and the “Communication” score (− 3.863 points vs − 1.564 points in females). Safinamide was shown to improve PD symptoms and quality of life in both male and female Chinese patients. Possible differences in the response between genders need to be further studied in larger and different ethnic populations.

## Introduction

Parkinson’s disease (PD) is characterized by classical motor features such as slowness of movements, muscular stiffness, tremor and gait impairment. With the progression of the disease some non-motor symptoms appear, such as autonomic dysfunction, psychiatric symptoms, chronic pain, cognitive deterioration, sleep disorders and fatigue^1^. Current therapies are mainly symptomatic. Levodopa (l-dopa) is considered the “gold standard” treatment for PD, however, after some years, the response to l-dopa doses is reduced and patients experience motor complications^2^. In vitro studies suggest that several neurotransmitters, other than dopamine, are involved in the control of motor symptoms and the development of dyskinesia^3^. Glutamate in particular, plays a key role in the pathophysiology of PD and in the emergence of motor complications^4^. Targeting nondopaminergic pathways could be a complementary approach to standard medications^5^.

Safinamide is a multimodal drug with a unique dual mechanism of action (MoA), dopaminergic and non-dopaminergic, that includes reversible monoamine oxidase-B inhibition and glutamate modulation^6–9^. The glutamatergic MoA is different from that of amantadine: safinamide inhibits the excessive release of glutamate through the sodium channels blockade, while amantadine is a NMDA receptors antagonist^10^. Results from pivotal studies in PD patients showed that safinamide, as add-on to levodopa, improves motor symptoms and motor complications maintaining the benefits in the long-term, and significantly increases ON time and decreases OFF time without increasing the risk to develop troublesome dyskinesia^11–14^. Moreover, safinamide was able to improve some non-motor symptoms such as chronic pain, mood deteriorations and sleep problems^15–20^.

The epidemiology and the clinical manifestations of PD are different between genders. Prevalence is higher in men who present more severe rigidity and gait problems, while women have generally a tremor dominant and less severe phenotype, possibly due to the different hormonal levels. Women experience more frequently motor complications and psychiatric disorders, with a significant social impact, while men have often a more rapid motor deterioration. Finally, women have a higher incidence of drug-related adverse events^21,22^. There is the need to implement new experimental strategies that integrate the concepts of sex and gender and make possible to improve the efficacy and tolerability of drug treatments in the two genders^23^.

This paper describes the results of new additional analyses in males and females Chinese PD subjects of the XINDI trial.

## Materials and methods

### Study design and study population

XINDI (NCT03881371) was a Phase III, double-blind, Chinese multicenter study with 305 patients treated with safinamide or placebo, as add-on to levodopa, for 16 weeks. Patients were eligible if ≥ 18 years, with idiopathic PD > 3-year duration^24^, Hoehn and Yahr (H&Y) stage 1–4^25^ and daily OFF time ≥ 1.5 h. The efficacy will be assessed by the changes in “OFF” and “ON” time from the 24-h patient diary, the Unified Parkinson’s Disease Rating Scale (UPDRS) and the Parkinson’s Disease Questionnaire-39 items (PDQ-39). The protocol and its amendments and the patients’ materials were approved by local Ethics Committees and by the National Health Authority. The study was conducted in compliance with the last version of the Declaration of Helsinki and the Good Clinical Practices^26^ and after the signature of a written informed consent by the patients. The confidentiality data of the subjects were protected according to the applicable data protection laws. Full details of the study and the results in the overall population have been published by Qianqian et al.^27^ and are also available in clinicaltrials.gov (NCT03881371).

### Data source and measurements

Patients’ data were recorded in electronic standardized case report forms (eCRFs) according to Good Automated Manufacturing Practice version 5 (GAMP5)^28^ and were checked to correspond to those registered in the official hospital files. Demographic data were retrieved during the baseline visit from the patient’s history and hospital clinical records. Medications were coded using the World Health Organization-Drug Dictionary (WHO-DD)^29^ and the adverse events (AEs) with the Medical Dictionary for Regulatory Activities (MedDRA) version 23.1^30^ on the basis of self-reported symptoms, and instrumental examinations. Serious adverse events (SAEs) were classified according to common definitions. All AEs and all SAEs were followed up until they were resolved.

Patients completed a 24-h home diary in the two days before each visit to track their OFF and ON time^31^. Patients were considered to be in ON when the medication is working and in OFF when the benefit abates. Dyskinesia was described as unexpected involuntary movements. The activities of daily living (ADL) and the motor symptoms (including the cardinal symptoms of PD) were evaluated during ON time with the UPDRS part II and III, respectively^32^. Patients’ quality of life (QoL) was assessed through the PDQ-39^33^.

### Statistical methods

All statistical analyses and data tabulations were produced using SAS^®^ for Windows release 9.4. All tests were two-sided and performed at the significance nominal level of α = 0.05. The primary study objective has been assessed by testing the superiority of safinamide compared to placebo in the “Full Analysis Set” (FAS) population, comprising all patients who provided informed consent, were randomized and received at least 1 dose or partial dose of the study drugs. Efficacy endpoints were summarized by arithmetic means, standard deviations, medians quartiles, minima and maxima and 95% confidence intervals. Counts and percentages were reported with the latest computed based on the numbers of patients with non-missing observations. Potential gender differences for the efficacy data were analyzed using a General Linear Model with the changes from baseline at the end of the study as dependent variable (response) and with baseline, treatment, gender, and treatment-by-gender interaction as independent variables (covariates). In case of statistical significance of the treatment-by-gender interaction^34^, the hypothesis of homogeneity of the response to safinamide across gender will be rejected and this implies that the statistical comparisons between safinamide and placebo will have to be carried out separately in males and females. Conversely, if the treatment-by-gender interaction is not significant, it can be concluded that the efficacy of safinamide is likely to be the same in both genders and therefore a statistical analysis pooling together males and females is appropriate. The incidence of adverse events in the two genders were analyzed using Fisher’s exact test.

### Ethics statement

This study involving human participants was reviewed and approved by the Independent Ethics Committees of the hospitals and the Chinese Health Authority and was conducted according to the ethical standards of the institutional and/or national research committee and the Declaration of Helsinki. The patients provided their written informed consent to participate in this study. The list of Ethics Committee is the following:

- Neurology Department, Shanghai Jiao Tong University School of Medicine, Ruijin Hospital, Shanghai, China
- Neurology Department, West China Hospital of Sichuan University, Chengdu, China
- Neurology Department, Sun Yat-sen Memorial Hospital, Guangzhou, China
- Neurology Department, The second affiliated hospital of Soochow University, Suzhou, China
- Neurology Department, Shanghai General Hospital, Shanghai, China
- Neurology Department, The Affiliated Hospital of Xuzhou Medical University, Xuzhou, China
- Neurology Department, Renmin Hospital of Wuhan University, Wuhan, China
- Neurology Department, The First Affiliated Hospital of Guangzhou Medical University, Guangzhou, China
- Neurology Department, Sir Run Run Shaw Hospital, Zhejiang University, Hangzhou, China
- Neurology Department, Sichuan Provincial People's Hospital, Chengdu, China
- Neurology Department, Chongqing Three Gorges Central Hospital, Chongqing, China
- Neurology Department, The Second Affiliated Hospital of Nanchang University, Nanchang, China
- Neurology Department, Wenzhou Medical College-The First Affiliated Hospital, Wenzhou, China
- Neurology Department, The First Hospital of Shanxi Medical University, Taiyuan, China
- Neurology Department, Beijing Friendship Hospital, Beijing, China
- Neurology Department, Baotou City Central Hospital, Baotou, China
- Neurology Department, Tianjin Union Medicine Center, Tianjin, China
- Neurology Department, The Third Hospital of Hebei Medical University, Shijiazhuang, China
- Neurology Department, The First Bethune Hospital of Jilin University, Jilin, China
- Neurology Department, The Affiliated Hospital of Guiyang Medical College, Guiyang, China
- Neurology Department, Tongji Hospital of Tongji University, Shanghai, China
- Neurology Department, Beijing Tiantan Hospital Affiliated to Capital Medical University, Beijing, China
- Neurology Department, Qilu Hospital of Shandong University, Jinan, China
- Neurology Department, The Second Affiliated Hospital of Zhejiang University, Hangzhou, China
- Neurology Department, Fujian Medical University Union Hospital, Fuzhou, China
- Neurology Department, Nanjing Drum Tower Hospital, Nanjing, China
- Neurology Department, Shanghai Ninth People's Hospital, Shanghai, China
- Neurology Department, The Third Xiangya Hospital of Central South University, Changsha, China
- Neurology Department, Zhengzhou, ChinaThe First Affiliated Hospital of Zhengzhou University
- Neurology Department, Daqing Oilfield General Hospital, Daqing, China
- Neurology Department, The First Affiliated Hospital of Baotou Medical University, Baotou, China
- Neurology Department, Guangzhou First People's Hospital, Guangzhou, China.

## Results

### Demography

Out of the 305 patients, 128 (42%) were women and 177 (58%) men (Table 1). There were no differences at baseline as for age, disease duration, H&Y stages, UPDRS scores, PDQ-39 score, levodopa dose, levodopa equivalent dose (LEDD) and the concomitant anti-parkinsonian drugs.

**Table 1:** Baseline patients’ overview according to gender.

		All population (n = 305)	Females (n = 128)	Males (n = 177)	p-value
Age at enrollment (years)	Mean (SD)	61.5 (9.2)	69.4 (9.4)	67.8 (9.7)	0.6471
Race (n, %)	Chinese	305 (100.0%)	128 (100.0%)	177 (100.0%)	–
Diagnosis (n, %)	Idiopathic PD	305 (100.0%)	128 (100.0%)	177 (100.0%)	–
Disease duration (years)	Mean (SD)	8.4 (5.0)	8.5 (4.9)	8.3 (4.8)	0.5937
Hoehn and Yahr stage (n, %)	1	11 (3.6%)	7 (5.5%)	4 (2.3%)	0.1919
	1.5	16 (5.2%)	8 (6.3%)	8 (4.5%)	
	2	137 (44.9%)	56 (43.8%)	81 (45.8%)	
	2.5	56 (18.4%)	18 (14.1%)	38 (21.5%)	
	3	78 (25.6%)	34 (26.6%)	44 (24.9%)	
	4	7 (2.3%)	5 (3.9%)	2 (1.1%)	
Total daily OFF time (h)	Mean (SD)	5.7 (2.9)	5.7 (2.8)	5.8 (3.0)	0.8564
Total daily ON time (h)	Mean (SD)	10.2 (2.9)	10.2 (3.0)	10.1 (2.9)	0.8595
Total daily ON time with no/non-troublesome dyskinesia (h)	Mean (SD)	9.7 (2.8)	9.9 (2.7)	9.6 (2.8)	0.4761
UPDRS total score (ON phase)	Mean (SD)	46.0 (18.3)	44.4 (17.9)	47.1 (18.5)	0.2062
UPDRS part II score (ON phase)	Mean (SD)	12.0 (5.6)	11.7 (6.0)	12.2 (5.2)	0.4991
UPDRS part III score (ON phase)	Mean (SD)	27.1 (12.9)	25.4 (11.9)	28.2 (13.5)	0.0649
PDQ-39 summary of index score	Mean (SD)	24.7 (13.0)	26.4 (13.5)	23.5 (12.5)	0.0600
Total daily levodopa dose (mg)	Mean (SD)	510.0 (185.0)	506.5 (210.0)	518.5 (200.0)	0.6200
Levodopa equivalent dose (mg)	Mean (SD)	800.0 (340.0)	786.5 (354.0)	810.5 (330.0)	0.4800
Concomitant antiparkinson drugs	Levodopa	305 (100.0%)	128 (100.0%)	177 (100.0%)	–
	Pramipexole	155 (100.0%)	66 (51.5%)	89 (50.2%)	
	Entacapone	110 (100.0%)	45 (35.1%)	65 (36.7%)	
	Amantadine	98 (100.0%)	42 (32.8%)	56 (31.6%)	
	Anticholinergics	42 (100.0%)	17 (13.2%)	25 (14.1%)	

Percentages (%) were computed by column.

*n* number of patients, *SD* Standard Deviation, *h* hours, *UPDRS* Unified Parkinsons’ Disease Rating Scale, *PDQ-39* Parkinson’s Disease Questionnaire-39 items, *mg* milligrams.

### Levodopa and levodopa equivalent dose

The mean l-dopa dose at baseline was 510 mg/day (± 185 mg) and the mean dose at the end of the study was 505 mg/day (± 190 mg). The mean LEDD at baseline was 800 mg/day (± 340 mg) and the mean LEDD at the end of the study was 786 mg/day (± 360 mg). There were no differences between the genders at follow-up regarding the l-dopa and LEDD doses (l-dopa: 500 ± 215 mg/day in women, 512 ± 205 mg/day in men; LEDD: 780 ± 360 mg/day in women, 800 ± 350 mg/day in men).

### Efficacy

Changes from baseline to week 16 in the efficacy parameters, comparing safinamide to placebo, are reported in Tables 2 and 3. At the end of the study, improvements were seen in favor of safinamide in both genders for all parameters analyzed, with statistically significant results for total daily OFF time (p = 0.0007), total daily ON time (p = 0.0036), ON time with no/non-troublesome dyskinesia (p = 0.0018) and UPDRS [total score (p < 0.0001), part II (p = 0.0003) and part III (p < 0.0001)]. The improvements in the UPDRS total score and in the UPDRS part III (motor examination) score were not only statistically but also clinically significant according to Shulman et al.^35^. The p-value for the “treatment-by-gender” interaction^36^ was non-significant for all parameters, confirming the homogeneity of treatment effects despite some differences between males and females. In particular, even if not statistically significant, we found that the changes from baseline at week 16 were > 50% higher in the females compared to males for the total daily OFF time (− 1.149 h vs − 0.764 h in males), the total daily ON time (1.283 h vs 0.441 h in males), the UPDRS total score (− 8.300 points vs − 5.253 points in males) and the UPDRS part II score (− 2.574 points vs − 1.016 points in males) (Table 2).

**Table 2:** Week 16: changes from baseline of the efficacy parameters according to gender.

Parameter	Gender	Difference safinamide-placebo, LS mean (SE)	95% CI	p-value^†^ (entire cohort as compared to baseline)
Mean total daily OFF time (h)	F	− 1.149 (0.426)	− 1.988, − 0.310	**0.0007**
	M	− 0.764 (0.367)	− 1.476, − 0.052	
Mean total daily ON time (h)	F	1.283 (0.448)	0.400, 2.165	**0.0036**
	M	0.441 (0.380)	− 0.307, 1.189	
Mean total daily ON time with no/non-troublesome dyskinesia (h)	F	1.107 (0.481)	0.159, 2.054	**0.0018**
	M	0.885 (0.408)	0.081, 1.689	
UPDRS total score	F	− 8.300 (2.215)	− 12.659, − 3.941	**< 0.0001**
	M	− 5.253 (1.877)	− 8.947, − 1.558	
UPDRS part II score	F	− 2.574 (0.743)	− 4.036, − 1.110	**0.0003**
	M	− 1.016 (0.630)	− 2.257, 0.224	
UPDRS part III score	F	− 4.985 (1.511)	− 7.959, − 2.010	**< 0.0001**
	M	− 3.933 (1.281)	− 6.453, − 1.411	
PDQ-39 summary of index score	F	− 4.305 (1.788)	− 7.824, − 0.784	**0.0014**
	M	− 3.253 (1.515)	− 6.235, − 0.270	
PDQ-39 mobility score	F	− 4.961 (2.501)	− 9.883, − 0.038	**0.0005**
	M	− 6.523 (2.122)	− 10.699, − 2.347	
PDQ-39 ADL score	F	− 6.965 (2.668)	− 12.215, − 1.714	**0.0003**
	M	− 5.772 (2.263)	− 10.226, − 1.317	
PDQ-39 emotional well-being score	F	− 6.243 (2.719)	− 11.594, − 0.891	**0.0035**
	M	− 4.203 (2.291)	− 8.712, 0.305	
PDQ-39 stigma score	F	− 6.185 (3.075)	− 12.236, − 0.133	**0.0063**
	M	− 4.913 (2.599)	− 10.028, 0.202	
PDQ-39 social support score	F	− 2.452 (2.233)	− 6.848, 1.943	0.112
	M	− 2.210 (1.892)	− 5.934, 1.513	
PDQ-39 cognition score	F	1.253 (2.362)	− 3.396, 5.902	0.4358
	M	1.165 (2.004)	− 2.780, 5.109	
PDQ-39 communication score	F	− 1.564 (2.681)	− 6.840, 3.712	0.1239
	M	− 3.863 (2.265)	− 8.321, 0.594	
PDQ-39 bodily discomfort score	F	− 5.196 (2.685)	− 10.481, 0.088	0.2438
	M	1.099 (2.272)	− 3.372, 5.570	

^†^The p-value for the comparison of Safinamide versus Placebo was calculated pooling together males and female because the treatment-by-gender interaction was not statistically significant. Significant values are in bold.

*LS* Least Squares, *SE* Standard Error, *CI* Confidence Interval, *h* hours, *UPDRS* Unified Parkinson’s Disease Rating Scale, *PDQ-39* Parkinson’s Disease Questionnaire-39 items, *ADL* Activities of Daily Living.

**Table 3:** Week 16: changes from baseline of the cardinal motor symptoms scores according to gender.

Parameter	Gender	Difference safinamide-placebo, LS mean (SE)	95% CI	p-value^†^ (entire cohort as compared to baseline)
Tremor	F	− 1.067 (0.487)	− 2.028, − 0.106	**0.005**
	M	− 0.746 (0.412)	− 1.557, − 0.066	
Bradykinesia	F	− 1.559 (0.707)	− 2.951, − 0.166	**0.0013**
	M	− 1.446 (0.597)	− 2.622, − 0.270	
Rigidity	F	− 0.984 (0.526)	− 2.020, 0.052	**0.0016**
	M	− 1.227 (0.445)	− 2.104, − 0.348	
PIGD	F	− 0.241 (0.382)	− 0.994, 0.512	0.1535
	M	− 0.478 (0.324)	− 1.117, 0.160	

^†^The p-value for the comparison of Safinamide versus Placebo was calculated irrespective of the gender because the treatment-by-gender interaction was not statistically significant. Significant values are in bold.

*LS* Least Squares, *SE* Standard Error, *CI* Confidence Interval, *PIGD* Postural Instability Gait Disorder.

**Figure 1:**
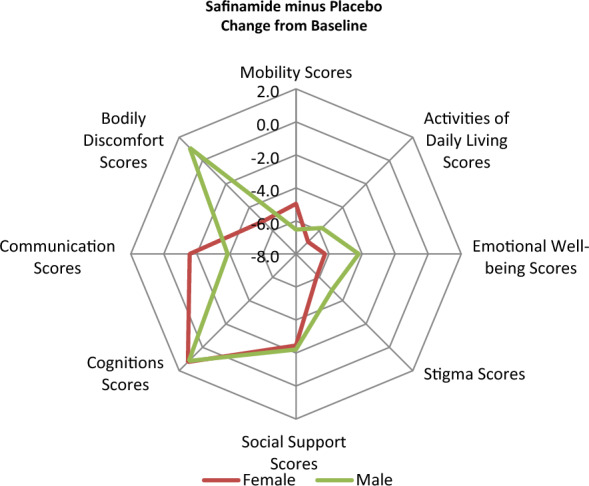
Synoptic diagram showing the changes from baseline at week 16 in the PDQ-39 domains’ scores according to gender.

**Figure 2:**
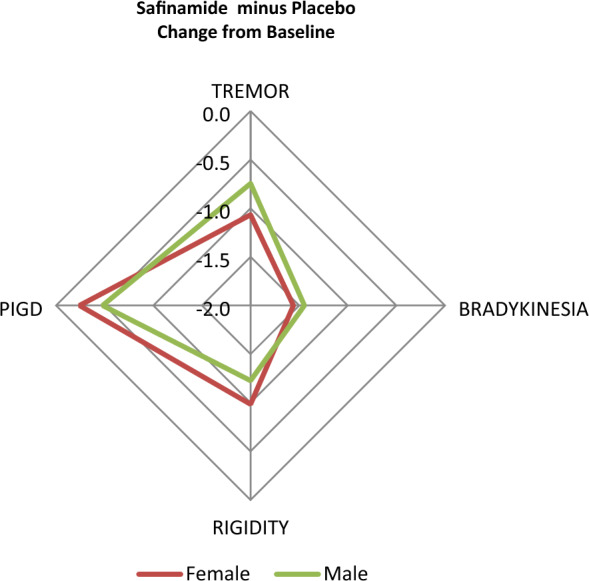
Synoptic diagram showing the changes from baseline at week 16 in the cardinal motor symptoms scores according to gender.

Safinamide, compared to placebo, significantly improved also the PDQ-39 Summary of Index score (p = 0.0014), the subscales scores for Mobility (p = 0.0005), ADL (p = 0.0003), Emotional well-being (p = 0.0035) and Stigma (p = 0.0063) (Table 2 and Fig. 1), and three cardinal symptoms: tremor (p = 0.005), bradykinesia (p = 0.0013) and rigidity (p = 0.0016) (Table 3 and Fig. 2). The changes from baseline at week 16 were higher in the females compared to males in the “ADL” domain (− 6.965 points vs − 5.772 points in males), the “Emotional well-being” domain (− 6.243 points vs − 4.203 in males), the “Stigma” domain (− 6.185 points vs − 4.913 points in males) and the “Bodily discomfort” domain (− 5.196 points vs 1.099 points in males), and higher in males in the “Mobility” score (− 6.523 points vs − 4.961 points in females) and the “Communication” score (− 3.863 points vs − 1.564 points in females) (Table 2 and Fig. 1).

Stratifications according to the administration of baseline medications as add-on to levodopa other than safinamide or placebo were not performed since concomitant multiple adjunctive treatments were administered and subgroups partly overlapped.

### Adverse events and serious adverse events

As reported in Table 4*,* during observation 82 (64.0%) female patients and 96 (54.0%) male patients experienced at least one adverse event (AE). This difference was not statistically significant. The majority of AEs were rated as mild or moderate. The most frequent AE was dizziness, with a slightly higher prevalence in females (11.7%) compared to males (8.4%). Other AEs with a frequency ≥ 3% of the total number of events were dyskinesia (7.8% in women vs 7.9% in men), nausea (7.0% in women vs 2.8% in men), back pain (5.4% in women vs 4.5% in men) and constipation (5.4% in women vs 3.9% in men). There were no statistically significant differences between genders and none of the above AEs was considered related to study treatment by the clinicians. Serious adverse events (SAEs) were rare and occurred in 3.9% of women and 4.5% of men: this difference was not statistically significant. All adverse events and SAEs were completed resolved at the end of the study. The analyses of laboratory examinations, vital signs, and electrocardiograms did not reveal any significant difference between males and females.

**Table 4:** Summary of adverse events according to gender.

	Females (n = 128)	Males (n = 177)	p-value^†^
Any AE	82 (64.0%)	96 (54.2%)	0.0996
Dizziness	15 (11.7%)	15 (8.4%)	0.4363
Dyskinesia	10 (7.8%)	14 (7.9%)	1.0000
Nausea	9 (7.0%)	5 (2.8%)	0.0998
Back pain	7 (5.4%)	8 (4.5%)	0.7909
Constipation	7 (5.4%)	7 (3.9%)	0.5860

Each subject is counted at most once within each primary system organ class and preferred term. Adverse events were classified according to Medical Dictionary for Regulatory Activities (MedDRA) version 23.1. Percentages are calculated on the number of subjects in the safety analysis set by investigational product.

*AE* adverse event, *n* number of subjects, *%* percentages of subjects.

^†^Fisher’s Exact Test.

## Discussion

This is the first publication that analyzes the potential sex differences in term of drug efficacy and safety in Chinese PD patients.

Motor fluctuations are defined as a rapid transition between good and poor response to medications. They are indicated by PD patients as the most disabling disease feature, even worse than dyskinesia. About 50% of subjects develop fluctuations after 2 years of disease, and this percentage increase up to 80% after 5 years, becoming more intense and unpredictable, with a significant economic burden^37,38^.

Safinamide, compared to placebo, significantly reduced OFF time, increased ON time (total and without dyskinesia) in both genders, therefore indicating that may also improve the quality of the ON time. This effect might be explained by the activity of safinamide on glutamate modulation^39^. Despite some data were suggestive of a better efficacy of safinamide in women, we did not find a significant treatment-by gender interaction, maybe due to the post-hoc design and the sample not large enough. However, gender differences in the effects of safinamide could be relevant especially for women, because they are known to develop more frequently than men motor fluctuations, and in particular wearing-off phenomena^7,9^. Moreover, women present a “brittle” response to l-dopa compared with men; wearing-off are associated with levodopa plasma levels and their stabilization may contribute to attenuate this complication^40^. In this study the LEDD did not change with safinamide treatment, confirming that a stable level of dose has been reached.

Safinamide improved three out of four PD cardinal symptoms except PIGD (postural instability gait disorder), which is known to be less responsive to drug treatments. In particular, safinamide reduced tremor in females and rigidity in males, the two peculiar gender features of PD^8^.

Picillo et al.^41^ found that men reported greater decline in daily motor activities, but this heterogeneity does not appear when motor assessment is evaluated by clinicians. Our study confirmed these findings and the improvements in the UPDRS motor scores were also clinically significant, despite optimized anti-PD therapy.

Consequently, there was an improvement in patients’ quality of life, as reflected by the PDQ-39 scale, a validated disease-specific questionnaire^42^. There is an association between sex and Qol which is generally worse in females due to psychological and social factors^43^. As described in the literature, “Emotional well-being”, “Stigma” and “Bodily discomfort” are the PDQ-39 domains with a greater severity in women while “Communication” is worse in men^44,45^. Balash et al.^46^ determined that also PDQ-39 SI scores are generally worse in women with greater “Emotional” and “Pain” items compared to men, while “Cognition” and “Communication” scores are more deteriorated in males.

These findings have been confirmed in our study where the improvements after safinamide treatment were higher in females in the socio-emotional domains (“Activities of daily living”, “Emotional well-being”, “Stigma” and “Bodily discomfort”), and in males in the physical-functioning domains (“Mobility and Communication”) (Fig. 2). The subscales “Emotional well-being” and “Bodily discomfort” reflect mood deterioration and pain, respectively. Depression and pain are known to be correlate and have a mutual and independent relationship with caregivers’ burden^47^. The positive results obtained with safinamide in these domains might be explained by its modulation of glutamate hyperactivity that is a common pathophysiologic mechanism of motor fluctuations, mood and pain^48–50^.

There are some limitations that should be considered in the interpretation of these data, in particular the sample size limitation and the trial design not considering an active comparator arm and thus preventing a direct comparison with other PD drugs. Moreover, this is a post-hoc analysis and the objectives were not predefined.

## Conclusions

Gender differences have been acknowledged to be an important determinant in the clinical manifestation of PD and in the response to the antiparkinsonian drugs, nevertheless no data are available in Chinese patients.

Our additional analyses of the XINDI study have shown that safinamide, compared to placebo, was associated with improvements in both genders, with some gender differences in the response.

Further studies are needed to investigate the effects of safinamide between genders in the real life and in different ethnic populations.

## Data Availability

The raw data supporting the conclusions of this article will be made available by the corresponding author, without undue reservation.
